# Augmenting Pentose Utilization and Ethanol Production of Native *Saccharomyces cerevisiae* LN Using Medium Engineering and Response Surface Methodology

**DOI:** 10.3389/fbioe.2018.00132

**Published:** 2018-09-24

**Authors:** Shalley Sharma, Eldho Varghese, Anju Arora, K.N. Singh, Surender Singh, Lata Nain, Debarati Paul

**Affiliations:** ^1^Division of Microbiology, ICAR-Indian Agricultural Research Institute, New Delhi, India; ^2^ICAR-Indian Agricultural Statistics Research Institute, New Delhi, India; ^3^Amity Institute of Biotechnology, Amity University, Noida, India

**Keywords:** *Saccharomyces cerevisiae*, co-fermentation, inhibitors, hydrolysates, response surface methodology, optimization

## Abstract

Economics of ethanol production from lignocellulosic biomass depends on complete utilization of constituent carbohydrates and efficient fermentation of mixed sugars present in biomass hydrolysates. *Saccharomyces cerevisiae*, the commercial strain for ethanol production uses only glucose while pentoses remain unused. Recombinant strains capable of utilizing pentoses have been engineered but with limited success. Recently, presence of endogenous pentose assimilation pathway in *S. cerevisiae* was reported. On the contrary, evolutionary engineering of native xylose assimilating strains is promising approach. In this study, a native strain *S. cerevisiae* LN, isolated from fruit juice, was found to be capable of xylose assimilation and mixed sugar fermentation. Upon supplementation with yeast extract and peptone, glucose (10%) fermentation efficiency was 78% with ~90% sugar consumption. Medium engineering augmented mixed sugars (5% glucose + 5% xylose) fermentation efficiency to ~50 and 1.6% ethanol yield was obtained with concomitant sugar consumption ~60%. Statistical optimization of input variables Glucose (5.36%), Xylose (3.30%), YE (0.36%), and peptone (0.25%) with Response surface methodology led to improved sugar consumption (74.33%) and 2.36% ethanol within 84 h. Specific activities of Xylose Reductase and Xylitol Dehydrogenase exhibited by *S. cerevisiae* LN were relatively low. Their ratio indicated metabolism diverted toward ethanol than xylitol and other byproducts. Strain was tolerant to concentrations of HMF, furfural and acetic acid commonly encountered in biomass hydrolysates. Thus, genetic setup for xylose assimilation in *S. cerevisiae* LN is not merely artifact of xylose metabolizing pathway and can be augmented by adaptive evolution. This strain showed potential for commercial exploitation.

## Introduction

Second generation bioethanol, produced by fermentation of sugars present in lignocellulosic biomass is a promising alternative fuel, due to the declining availability of fossil fuels and their negative impact on the environment (Cai et al., 2012; Lin et al., 2012; Yadav et al., 2018). *Saccharomyces cerevisiae*, widely used in starch and sucrose based 1st generation bioethanol production, is believed to be the most appropriate candidate for lignocellulosic bioethanol production due to its highly efficient hexose fermentation under anaerobic conditions, high tolerance to both ethanol and inhibitory compounds present in lignocellulosic hydrolysates. Although some bacteria, such as Zymomonas mobilis and genetically modified *Escherichia coli*, are capable of fermenting diverse sugars (Dien et al., 2003), the yeast *S. cerevisiae* is still the preferred organism for industrial production of ethanol because of its high ethanol tolerance, GRAS status, tolerance of low pH, as well as resistance to bacteriophage infection making it particularly relevant in large industrial processes (Albergaria and Arneborg, 2016; Moysés et al., 2016). Further improvements in product yields and productivity have come by strain improvement and process optimization of starch and sucrose based bioethanol. However, scenario for production of 2nd generation bioethanol from lignocellulosic biomass is different and is beset with challenges like highly recalcitrant feedstock, inefficient enzymatic conversion of polymeric sugars to simple sugars, presence of inhibitors and diverse sugars in hydrolysates including xylose, which S. cerevisiae cannot use or ferment. The major fermentable sugars in lignocellulosic biomass hydrolysates are D-glucose and D-xylose. To obtain an economically feasible industrial process for bioethanol production, it is imperative to efficiently ferment both the sugars into ethanol (de Sales et al., 2015).

Numerous strategies have been applied to enable *S. cerevisiae* to utilize and co-ferment mixture of sugars as complete utilization of both hexoses and pentoses will lead to higher ethanol yield and favorable economics. Traditionally the wild-type *S. cerevisiae* has been thought to be incapable of utilizing xylose, a pentose sugar, the second dominant sugar in lignocellulosic hydrolysates, for growth or fermentation. The most common approaches for conferring xylose utilization ability on *S. cerevisiae* have been to genetically engineer the strain and enhance expression of xylose reductase gene (XR), xylitol dehydrogenase (XDH) and xylulokinase (XK) genes (Ho et al., 1998; Vilela Lde et al., 2015; Feng et al., 2018). Expression of XR and XDH genes lead to xylitol end product. Introduction of xylose catabolic pathway into *S. cerevisiae* does not provide any solution to the efficient lignocellulosic bioethanol fermentation as single characteristic/phenotype is not controlled by a single gene or a single modification (Bailey, 1999). Many *S. cerevisiae* strains possess endogenous XK genes and can metabolize xylulose, a keto-isomer of xylose, into ethanol via pentose phosphate pathway. However, it was later observed that xylose is not efficiently transported into the cells. Numerous efforts have been made to link extracellular xylose with intracellular xylulose by equipping S. cerevisiae with heterologous transporters for xylose in addition to xylose metabolizing genes (Matsushika et al., 2009; Shin et al., 2015; Li et al., 2016). Therefore, a major focus in metabolic engineering has been to improve xylose assimilation in *S. cerevisiae*. But none of the efforts made so far has resulted in a strain of industrial significance and in spite of various metabolic engineering efforts for improved xylose fermentation, xylose consumption rate and ethanol yield do not match glucose uptake and fermentation, thus need further improvement.

It is, often, a major misconception that *S. cerevisiae* is unable to grow on xylose as sole carbon source. Xylose-positive strains have been previously reported (Wenger et al., 2010; Li et al., 2016). The study demonstrated that some strains of S. cerevisiae possess native xylose utilization capacity enabled by putative xylitol dehydrogenase (XDH) thus challenging the need for heterologous pathway engineering (Wenger et al., 2010). While other native xylose-utilizing organisms from other genera exist, they largely lack well-developed genetic tools for host engineering or exhibit low product and inhibitor tolerances (Blazeck and Alper, 2010). Therefore, emphasis should be given on uncovering and evolving the latent natural and innate xylose utilizing capabilities in *S. cerevisiae* strains (Wenger et al., 2010). These endogenous xylose utilizing pathways may be more compatible than the heterologous genes.

Furthermore, native *S. cerevisiae* lack high-affinity xylose transporters and instead rely on low affinity transport through glucose transporters, resulting in slower diauxic sugar consumption. Although pilot scale experiments have demonstrated high ethanol yields from the D-xylose present in plant biomass hydrolysates, strain robustness, especially with respect to tolerance to inhibitors present in hydrolysates, can still be improved further (Van Maris et al., 2007; Reider Apel et al., 2016). To overcome these barriers, adaptation and directed evolution are commonly employed strategies to significantly improve the rate of xylose fermentation from laboratory media (Sato et al., 2014).

In this study, a native strain of S. cerevisiae LN ITCC 8246 was found to weakly assimilate xylose as sole C source, and produce ethanol from mixed substrates (glucose + xylose). Medium engineering was employed to improve xylose utilization and enhance the fermentation efficiency. Parameters were optimized using response surface methodology. Tolerance of native *S. cerevisiae* strain toward concentrations of inhibitors, such as HMF, furfural, acetic acid and formic acid at concentrations typically encountered in biomass hydrolysates, was also studied to ascertain its potential for application in lignocellulosic ethanol fermentations.

## Materials and methods

### *S. cerevisiae* LN ITCC 8246, its substrate utilization and fermentation capability

*S. cerevisiae* strain LN ITCC 8246 was obtained from the culture collection of Division of Microbiology, Indian Agricultural Research Institute (IARI), India. It is a native isolate obtained from the rotten fruit (Nain and Rana, 1987). Culture was grown on MGYP (3 g L^−1^ Malt Extract, 10 g L^−1^ Glucose, 3 g L^−1^ Yeast Extract and 5 g L^−1^ Peptone) medium at 30°C and stored at 4°C.

*S*. *cerevisiae* LN was examined for the assimilation of different sugars using HiMedia sugar strips on qualitative basis. Incubation was carried out at 30°C for 48 h and observed for change in color of indicator dye. Xylose assimilation was also confirmed by observing growth of *S. cerevisiae* LN on plates containing minimal medium (1 g L^−1^ KH_2_PO_4_, 5 g L^−1^ MgSO_4_, 5 g L^−1^ (NH_4_)SO_4_, 1 g L^−1^ Yeast extract) and agar with 1% xylose as sole C source.

Xylose utilization and its fermentation to ethanol was also quantified. Organism was grown in 10 mL minimal medium with 2% xylose in 15 mL screw capped vials, incubated at 30°C for 72 h. 1 mL sample was taken at regular intervals. Culture samples were centrifuged at 8,000 rpm. Residual xylose in supernatants was estimated by DNSA using xylose standard (Miller, 1959) and ethanol was estimated by gas chromatography.

### Fermentation of glucose/xylose/mixed sugars

Fermentation experiments were carried out in fortified medium (1 g L^−1^ KH_2_PO_4_, 5 g L^−1^ MgSO_4_,5 g L^−1^ (NH_4_)SO_4_, 1 g L^−1^ Yeast extract) with 10% glucose or mixed sugars (glucose 5% + xylose 5%). Inoculum was prepared by growing culture in MGYP medium (24 h old culture, ~1.8 OD_660nm_) and added at 5% rate to 50 mL fortified medium with sugars in 100 mL Erlenmeyer flasks. Fermentation was carried out at 30°C for 144 h. Experiment was carried out in two phases with shaking at 150 rpm till 48 h and then static conditions were maintained till the end of the process. Samples were aseptically withdrawn at regular intervals of 24 h to determine sugars consumed and ethanol produced. They were analyzed using high performance liquid chromatography (HPLC). Fermentation efficiency was calculated as follows (McMillan, 1993):

(1) $$\begin{array}{r} \%\;Fermentation\;Efficiency=\left\{\frac{Actual\;Ethanol\;Yield\;\left(g\right)}{Theoretical\;Ethanol\;Yield\;\left(g\right)}\right\}X100 \end{array}$$

(2) $$\begin{array}{r} Theoretical\;Ethanol\;Yield\;\left(g\right)=\left\{Sugar\;Consumed\;\left(g\right)X\;0.511\right\} \end{array}$$

### Effect of medium engineering on fermentation efficiency

Minimal medium was supplemented with different concentrations of yeast extract (0.1, 0.5, and 1%), peptone (0.1 and 1%). Experimental conditions were maintained as previously described. Cultures were withdrawn periodically and processed for analyzing sugar consumption and ethanol production using HPLC.

### Optimization of sugar utilization and fermentation using response surface methodology (RSM)

#### One way and two way ANOVA: SAS

The data collected on mixed substrate fermentation and glucose fermentation by *S. cerevisiae* LN were subjected to two way analysis of variance (two-way ANOVA) and significant effects were noted. Critical difference (CD) with 95% significance level and SE_m_ were also worked out using SAS (version 9.4). Data collected for growth and fermentation on 2% xylose was analyzed through one way ANOVA, as there was only one dependent variable in it.

#### Response surface methodology

Response Surface Methodology (RSM) consists of the experimental strategy for exploring the relationship between the response variable and the input variables and to develop an appropriate approximating relationship between them. In present study, a second order response surface model was used as presented below:

(3) $$\begin{array}{rcl} f\left(x_{u}\right) & = & \;\beta_{0}+\;\sum_{i=1}^{5}\beta_{i}x_{iu}+\;\sum_{i=1}^{5}\beta_{ii}x_{iu}^{2}\\ +\;\sum_{i=1}^{4}\sum_{i^{\prime }=i+1}^{5}\beta_{ii}x_{iu}x_{i}^{{\;}^{\prime }}u+e_{u} \end{array}$$

where u = 1, 2, …, N, x_iu_ is the level of the i_th_ (i = 1, 2, …, 5) factor in the uth treatment combination, f(x_u_) denotes the response obtained from uth treatment combination and e_u_ is the random error associated with the uth observation that is independently and normally distributed with mean zero and common variance σ^2^, β_0_ is a constant, β_i_ is the ith linear regression coeffiient, β_ii_ is the ith quadratic regression coefficient and $\beta_{\text{ii}}^{{\;}^{\prime }}$ is the (i, i′)th interaction coefficient (Nath et al., 2015).

#### Response surface design

A five-level, five-factor, Central-Composite design under RSM was used to optimize the conditions of ethanol production and sugar consumption by *S. cerevisiae* LN under fermentation conditions using mixed substrates and complex medium supplemented with yeast extract and peptone. Two responses were selected to study the optimized conditions for fermentation. Responses were ethanol production and sugar consumption (glucose and xylose). Input parameters taken were xylose concentration, glucose concentration, yeast extract concentration, peptone concentration and time taken to ferment sugars (Table 1).

**Table 1:** Independent variables with coded levels and actual values for fitting response surface model.

**Independent variables**	**Units**	**Code levels**				
		−α	−**1**	**0**	+**1**	+α
Glucose	%	1.62	3	4	5	6.37
Xylose	%	0.46	1.5	2.25	3	4.03
Time	h	24.3	45	60	75	95.68
Yeast Extract	%	0.13	0.2	0.25	0.3	0.37
Peptone	%	0.13	0.2	0.25	0.3	0.37

Fermentation conditions were optimized by optimizing the fermentation medium (glucose, xylose, yeast extract and peptone concentrations) along with the fermentation time. The limits taken for the optimum fermentation conditions were: glucose (1.6–6.37%), xylose (0.46–4.03%), yeast extract (0.13–0.37%), peptone (0.13–0.37%), and time (24.3–95.68 h). These optimum conditions were determined by numerical optimization technique using Design Expert software. The main criteria for optimization was higher ethanol production and higher sugar consumption by the *Saccharomyces* strain. To achieve optimum conditions, satisfying the imposed criteria, the goals are combined into an overall composite function called the desirability function (Cavalaglio et al., 2016).

Second order response surface model was fitted to the data. Significant parameter estimates were identified. Response surface optimization and also the generation of CCD for the experiment were done using Design Expert Software (version 9.0).

### Xylose reductase and xylitol dehydrogenase enzymatic activities in *S. cerevisiae* LN ITCC 8246

*S. cerevisiae* LN was grown for 48 h on 2% xylose/2% glucose/2% (xylose + glucose) in minimal medium with shaking at 150 rpm at 30°C. After 48 h, cultures were centrifuged at 8,000 rpm for 10 min and supernatants were discarded. Pellet was processed for determining XR (xylose reductase) and XDH (xylitol dehydrogenase) activities in intracellular milieu.

XR activity was determined by washing the pellet obtained twice with 250 mM phosphate buffer, pH 7.0), sonicated (30% amplitude with a pulse of 20 s for 30 min, Hielscher Ultrasound Technology) in an ice jacket. The lysate was centrifuged at 8,000 rpm and supernatant was used as the crude enzyme extract. Two cocktails were prepared as shown in Table S1. Crude enzyme (50 μL) was added to the experimental vial and readings were taken at 340 nm for 3 min and the rate of change of OD was used to determine the enzyme activity (Yokoyama et al., 1995).

For XDH activity estimation, cell pellet was washed twice with Tris-Cl buffer (pH 8.6), sonicated (as above), and the lysate was then used as the crude enzyme extract. For this assay, two cocktails were prepared as shown in Table S2 into two separate cuvettes and kept on ice (Ikeuchi et al., 2000). 50 μL of crude enzyme was added to the experimental vial and measurement of the rate of change min^−1^ at 340 nm was considered as the XDH activity of *S. cerevisiae* LN. Protein concentration in extract was measured using BSA as standard. Specific activities of the enzymes were calculated based on protein concentrations.

### Inhibitor tolerance of native *S. cerevisiae* LN

Pretreatment techniques, applied for biomass deconstruction, generate byproducts inhibitory to microbial growth and fermentation. To check the suitability of this strain for bioethanol production from biomass hydrolysates, effect of different inhibitors on its growth was studied by growing *S. cerevisiae LN* in synthetic medium containing inhibitors at concentrations commonly encountered in biomass hydrolysates.

*S. cerevisiae* LN was grown in presence of HMF (0.5–2.0 g L^−1^) and furfural (0.25–0.65 g L^−1^) in minimal medium with 5% glucose + 2.5% xylose for 96 h. Growth was checked every 24 h by reading absorbance at 660 nm. Appropriate controls were maintained and growth was compared. Growth was also checked in presence of acetic acid (5–15 g L^−1^) and formic acid (3–11 g L^−1^) under similar conditions. All the experiments were carried out in triplicates.

### Analytical methods

Cultures harvested at regular intervals were centrifuged at 8,000 rpm for 10 min, filtered with 0.22 μm Nylon-66 HPLC syringe filters and subjected to chromatographic analysis by HPLC for ethanol and sugar estimation.

### High performance liquid chromatography

Samples were run on Aminex HPX-87H column (Bio-Rad, Hercules, CA, USA) at 65°C using 5 mM H_2_SO_4_ as mobile phase at 0.5 mL min^−1^ and measured with a ShodexRI-101 refraction index detector (Shoko Scientific Co. Ltd., Yokohama, Japan) as described (Arora et al., 2016).

## Results

### *S. cerevisiae* LN, its sugar utilization range and fermentation capability

In this study, *S. cerevisiae* LN ITCC 8246 (accession no. KF953906), a native strain isolated from rotten fruit was found to weakly grow on plates containing minimal medium with xylose as sole C source. It was also capable of utilizing different sugars including melibiose, maltose, sucrose, galactose and raffinose but could not utilize cellobiose, inositol, dulcitol and trehalose.

#### Glucose/xylose fermentation

Results (Table 2) showed that *S. cerevisiae* LN could utilize xylose and ferment it to ethanol with fairly high efficiency but its uptake was very less. It could utilize <20% of xylose provided in 72 h, indicating that it had metabolic machinery to ferment xylose to ethanol but not efficient transporters for efficient uptake of xylose.

**Table 2:** Growth, sugar consumption and ethanol production by *S. cerevisiae* LN on 2% xylose.

**Time (h)**	**Growth (OD_600 nm_)**	**Ethanol (%)**	**Sugars consumed (g L^−1^)**	**Fermentation efficiency (%)**	**Ethanol yield (g g^−1^)**
24	0.61	0.05 ± 0.18	2.13 ± 0.29	45.11 ± 0.16	0.23 ± 0.003
48	0.85	0.09 ± 0.01	3.74 ± 0.13	46.29 ± 0.46	0.24 ± 0.007
72	1.07	0.11 ± 0.41	3.61 ± 0.19	58.82 ± 0.59	0.30 ± 0.01
^*^SE_m_		0.03	1.11	7.96	0.04
^**^CD@5%		0.10	3.06	21.97	0.11

* SE_m_ denotes Standard error of mean;

** CD@5% denotes critical difference @ 5%.

In minimal medium containing 10% glucose and supplements i.e., YE or peptone, *S. cerevisiae* LN completely utilized sugar and showed highest fermentation efficiency ~77% at 120 h (Table S3). While glucose depletion was similar in all the treatments, ethanol yields, however, were varied and thus different fermentation efficiency. This is the consequence of nature and level of supplements added to the medium and time of incubation.

#### Medium engineering for enhanced mixed substrate utilization and fermentation

**Figure 1:**
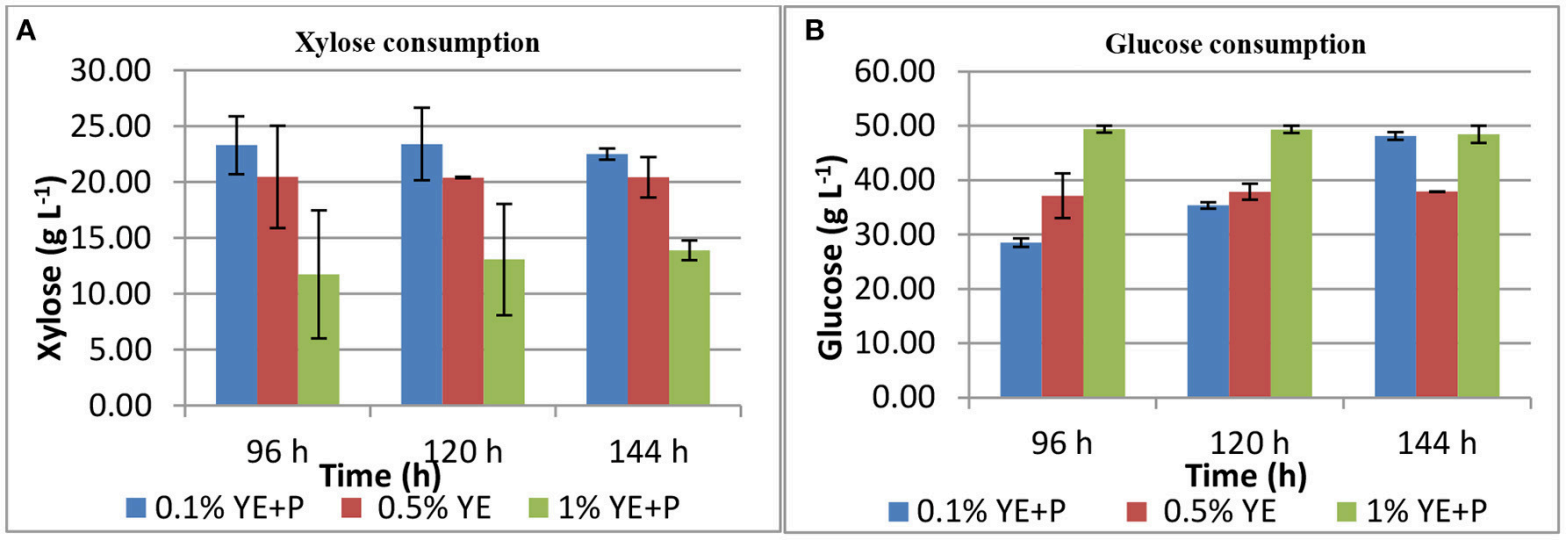
Xylose **(A)** and glucose **(B)** consumption during mixed substrate fermentation by *S. cerevisiae* LN.

*S. cerevisiae* LN, grown in minimal medium containing mixed substrates (5% glucose + 5% xylose), supplemented with different concentrations of rich organic components i.e., yeast extract, peptone, and their combination showed better growth, sugar utilization and ethanol production (Table 3). Highest fermentation efficiency of ~50% was obtained at 96 h with 1% yeast extract + 1% peptone and 61% concomitant sugar consumption with no further sugar depletion thereafter and highest ethanol yield of 0.25 g g−1. Also, at 0.5% YE supplementation mixed sugar consumption reached maximum at 96 h. It did not increase further on prolonged incubation. Whereas, at low level of supplementation (0.1% YE and Peptone), mixed sugar consumption gradually increased from 51 to 70% upon further incubation till 144 h. Figure 1 presents consumption pattern of individual sugars (glucose and xylose) by *S. cerevisiae* LN cultured on mixed sugars. Xylose consumption did not change significantly over prolonged incubation at different levels of supplementation. Glucose consumption on the other hand was much faster at high level of supplementation and was depleted within 96 h while it increased upon longer incubation at lower supplementation of YE and Peptone (0.1 and 0.5% YE). At lower supplementation level (0.1%), sugar depletion increased till 144 h reaching 71% with very low ethanol production. Xylose consumption was ~45% by 96 h at low levels of supplementation and did not increase with further incubation or higher supplementation levels. However, glucose consumption increased with incubation at low supplementation level (0.1% YE and peptone) while at higher supplementation levels glucose was depleted faster.

**Table 3:** Stimulation of mixed sugar consumption and fermentation efficiencies of *S. cerevisiae* LN upon supplementation of minimal medium with yeast extract and peptone.

**Treatment**	**Total sugar consumed (g L**^**−1**^**)**			**Mean**	**Fermentation efficiency (%)**			**Mean**
**Time (h)**	**96**	**120**	**144**		**96**	**120**	**144**	
0.1% (YE + P)^*^	51.81 ± 3.37	58.74 ± 3.83	70.61 ± 1.22	65.13	5.33 ± 3.39	16.11 ± 6.65	9.54 ± 7.01	10.73
0.5% YE	57.58 ± 8.68	58.27 ± 1.41	54.09 ± 1.75	56.63	22.58 ± 4.76	21.4 ± 1.25	27.14 ± 3.78	24.68
1% (YE + P)	61.12 ± 5.10	62.42 ± 4.33	62.32 ± 0.68	61.96	48.14 ± 11.72	42.35 ± 9.05	38.63 ± 1.27	43.56
Mean time	56.84	59.798	67.082		25.98	26.03	26.967	
SE(Time)	6.60	CD(Time)	14.93		SE(Time)	8.99	CD (Time)	20.35
SE(trt)	3.81	CD(trt)	8.62		SE(trt)	5.19	CD (trt)	11.75
SE(trt^*^Time)	3.81	CD (trt^*^Time)	8.62		SE(trt^*^Time)	5.19	CD (trt^*^Time)	11.75

* *(YE + P), Yeast extract + Peptone*.

Increase in glucose consumption (~60%) at low supplementation level (0.1% YE and peptone) and its depletion at higher supplementation levels revealed that glucose was taken up by the strain earlier while later on xylose was being consumed and ethanol produced.

Ethanol production at prolonged later hours was either increasing or consistent with the previous hours at all levels of supplemenation.

### Optimization of fermentation conditions using RSM

Observations from mix sugar fermentation experiment showed the need for optimizing input concentration of glucose, xylose, YE and peptone and also incubation time to get maximum utilization of mixed sugars and ethanol production with higher efficiency. Thus, on the basis of enhancement in fermentation efficiency by medium engineering, the supplementation conditions were optimized using response surface methodology. Response surface methodology allows improvisation and optimization of processes in which a response of interest is being influenced by several variables (Pandiyan et al., 2014).

As observed from the above experiments, conditions for optimization of fermentation were: glucose concentration, 1.6–6.3%; xylose concentration, 0.64–4.0%; peptone concentration, 0.13–0.37%; yeast extract concentration, 0.13–0.37%; time, 24–96 h. Minimal medium was used for fermentation experiments.

#### Ethanol production

Quantification of ethanol produced and the equation connecting ethanol with other input variables showed an overall significance at *p* ≤ 0.001. Individual parameter estimates, glucose concentration (A), xylose concentration (B), A^2^ are significant model terms. ANOVA for response surface quadratic model for ethanol production has *p*-value 0.000338, which makes this model significant. The response ethanol (R1) was found to be a best fitted model due to the dependency of the response on the input variables, such as glucose, xylose, time of fermentation, yeast extract, and peptone concentration.

Surface plots indicate that as the glucose concentration increases, ethanol production increases as well (Figures S1B,C). These plots also suggest that yeast extract and peptone concentrations have negligible effects on ethanol production (Figures S1D–F). Even in the presence of xylose, with time ethanol production slightly increased suggesting that this native strain can utilize and ferment xylose to some extent (Figure S1A).

#### Sugar consumption

The model best fitted for sugar consumption showed an overall significance at *p* ≤ 0.05. This is response sugar consumed (R^2^). There is only a 0.01% chance that a lack of fit F-value, this large (34,641.11) could occur due to noise.

Surface plots generated by the software (Figures S2A,B) suggested that sugar consumption increased with increasing individual concentrations of sugars but the most of the impact was due to glucose and then xylose, supplementation had least effect on the consumption which makes the whole process economic from industrial point of view.

A simultaneous Multi response optimization was performed on both the responses by maximizing the responses and optimum value for the input variables was obtained.

The best combination of process variables for the best set of response properties was: glucose concentration (5.36%), xylose (3.30%), fermentation time (84.55 h), yeast extract (0.36%) and peptone (0.25%). In order to verify the predictive capability of the model, optimum conditions were established by RSM and comparisons between predicted results and the practical values were done by experimental rechecking using those presumed optimal conditions. The predicted responses in terms of ethanol production and sugar consumption were 2.99 and 81.67%, respectively and the observed responses are presented in Table 4.

**Table 4:** Predicted and actual response values of optimized fermentation experiment for *S. cerevisiae* LN.

**Response**	**Predicted values**	**Observed values**
Ethanol produced (%)	2.99	2.36
Sugars consumed (%)	81.67	74.33

The results obtained suggested that the change in input variables (glucose, xylose, time, yeast extract and peptone) had a significant (*p* < 0.05) effect on ethanol production and sugar consumption. Therefore, it could be deduced that all the fermentation conditions including the concentration of sugars as well as peptone and yeast extract play a major role in the optimum fermentation of the substrates for efficient ethanol production. Under optimum conditions, the observed responses were 2.36% ethanol production and 74.33% total sugar consumption. There was an excellent agreement of the observed experimental values with the predicted values indicating the suitability of the models developed and the success of RSM in optimizing fermentation conditions. It was observed that after optimizing input concentration of hexose: pentose, native strain *S. cerevisiae* LN, consumed sugars very efficiently (74% of initial 8.66% total sugars) and utilized good amount of xylose (33%) provided in the medium.

Thus, in this RSM optimization protocol, glucose and xylose were provided in the ratio similar to that present in biomass hydrolysates. The organism was able to use ~75% of total sugars provided. Assuming that whole of glucose was utilized, organism depleted ~35% xylose and showed high fermentation efficiency. Therefore, it indicates at lower supplementation levels, ethanol production and overall sugar utilization increased along with xylose consumption.

### XR and XDH activities

For pentose metabolism in yeast, enzymes xylose reductase (XR) and xylitol dehydrogenase (XDH) play a major role. Although *S. cerevesiae* possess orthologous genes for XR and XDH, but still they are inefficient to grow on xylose as sole carbon source (Chang et al., 2007; Nielsen et al., 2015) suggesting the importance of transport, uptake, and ratio of these xylose catabolic enzymes and it should be further studied. Other factors like redox potential and cofactors play an important role in xylose metabolism.

In this study, *S. cerevesiae* LN showed low XR and XDH specific activities with xylose as sole C source and ratio of XR: XDH activity was also lowest Table 5. Depending upon the substrate, XR:XDH ratio for *S. cerevisiae* LN varied from 4.2, 3.8, 1.5. Lowest ratio in case of xylose as the substrate signified that most of the xylose consumed was diverted for ethanol production rather than xylitol.

**Table 5:** XR and XDH sp. activities of *S. cerevisiae* on different substrates.

**Enzyme extract (different substrates)**	**Sp. Activity (U mg**^**−1**^ **protein)**		**Ratio XR:XDH**
	**XR**	**XDH**	
2% Glucose+ 2% Xylose	0.18	0.047	3.829
Xylose (2%)	0.0097	0.0066	1.469
Glucose (2%)	0.15	0.036	4.166

### Tolerance to inhibitors

**Figure 2:**
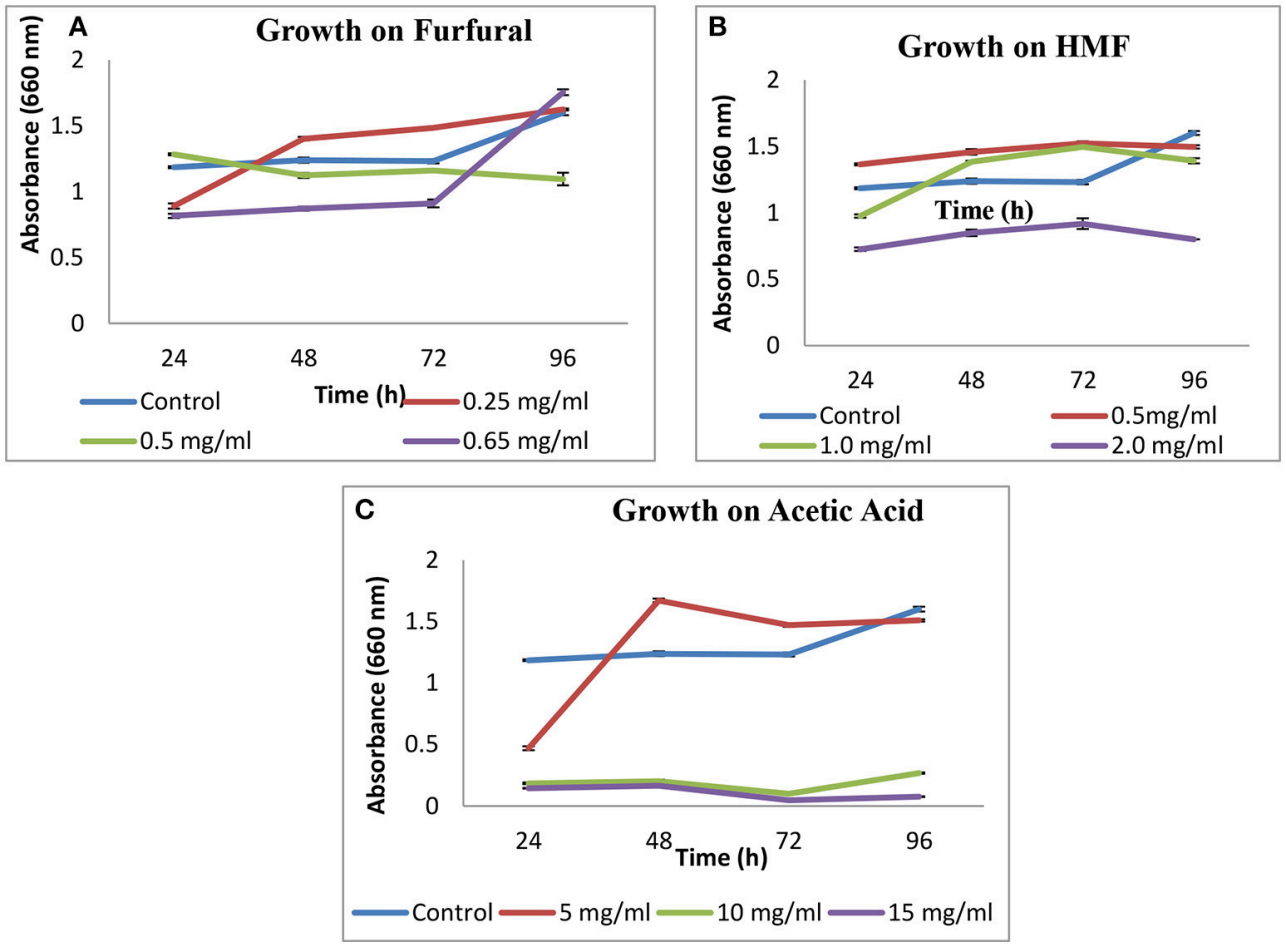
Effect of furfural **(A)**, HMF **(B)**, and acetic acid **(C)** on growth of *S. cerevisiae* LN.

Growth of *S. cerevisiae* strain LN was checked on HMF, furfural and acetic acid. On HMF and furfural, it showed good growth upto concentration 1 g L^−1^ and slightly decreased at 2 g L^−1^ as compared to control. On furfural growth pattern was good and similar to control till 0.5 g L^−1^. On increasing concentration to 0.65 g L^−1^ growth was lesser as compared to control till 72 h and increased thereafter at 96 h (Figure 2).

In case of acetic acid, a sudden rise in growth was observed after 72 h for 5 g L^−1^ concentration.

## Discussion

There are several reports on the co-utilization of hexoses and pentoses by engineered strains of *S. cerevisiae* Native *S. cerevisiae* strains have also been reported to weakly utilize xylose. This study focuses on the augmenting innate machinery and metabolism of native strain of *Saccharomyces* for mixed substrate fermentation. Innate mechanisms could be potentially augmented further through evolutionary engineering to reprogram the metabolic fluxes (Jansen et al., 2017).

*S. cerevisiae* LN ITCC 8246 a native strain weakly grew on minimal agar medium with xylose as sole C source and utilized a wide range of sugars. This strain grew well on paddy straw hydrolysates and produced ethanol (Arora et al., 2016).

Yeasts are capable of using diverse substrates and are infamous for their food spoiling abilities and are involved in spoiling foods with high concentration of sugars, such as honey, maple syrup, sugar cane, and confectionery (Scott, 1957; Ingram, 1958; ONISHI, 1963). Ability to utilize a diverse range of sugars depending upon the availability is highly significant and noteworthy as most of the industrial applications of *S. cerevisiae* rely on its ability to efficiently ferment sugars, even under fully aerobic conditions (Lagunas, 1979). Weak pentose utilization by *S. cerevisiae* strains (mostly wine yeasts) has been attributed to *XDH1* gene (Wenger et al., 2010). The xylose utilization capacity and ethanol fermentation efficiency is optimal at lower level of supplementation with organic supplements, Addition of peptone promotes growth and biomass production than fermentation and upon prolonged incubation ethanol produced can get converted to products like acetic acid. Growth and physiology of yeasts is profoundly affected by medium components including buffers and rich organic additives. It can be manipulated by medium supplementation and changing nutrients and other growth factors. Impact of cultivation media components and incubation time on growth and physiology of yeasts is reflected directly by their fermentation/product profile Thus, medium and process engineering should form an integral part of approaches for strain development (Hahn-Hägerdal et al., 2005).

**Figure 3:**
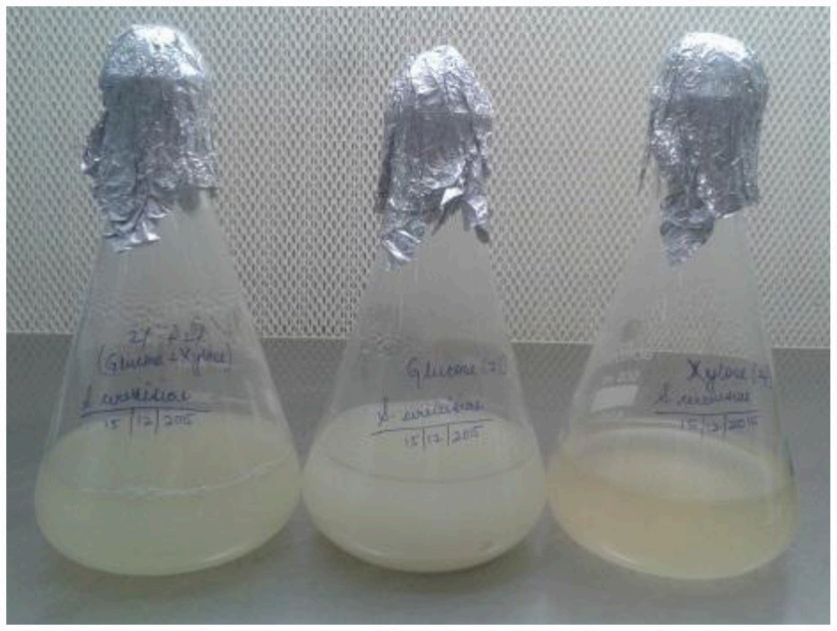
Growth of *S. cerevisiae* LN on xylose, glucose and mixed substrates in minimal medium.

During growth on mixed sugars *S. cerevisiae* LN utilized both glucose and xylose but xylose uptake was not as high as glucose (Figure 3). No xylose-specific transporters have been reported in *S. cerevisiae* and xylose is taken up in the cells via hexose transporters (Katahira et al., 2008), thus making xylose uptake slower. Ethanol production with prolonged incubation suggested ethanol production from xylose as glucose levels were already exhausted.

A study of xylose fermentation, carried out by recombinant *S. cerevisiae*, showed that sugar uptake occurred through *HXT2.6* gene and was fermented to ethanol in the first 10 h but from that point on, no ethanol was produced by the cells and the consumed xylose was reduced into xylitol, which accumulated in the media into the same levels as ethanol. During co-fermentation the rates of glucose consumption were practically not affected (de Sales et al., 2015).

Biomass hydrolysates usually contain higher amount of glucose than xylose as cellulose is predominant structural polysaccharide in most of lignocellulosic biomass. Therefore, ratio of glucose and xylose in growth medium becomes critical for optimum utilization of both sugars as they share common transporters for getting assimilated into the cells (Nijland et al., 2017). RSM is an important empirical procedure for optimizing input variables, such as nutrients for both defined and complex media to get maximal desired responses. This strategy has been adopted for rational optimization and maximum productivity thus getting favorable process economics (Myer and Montgomery, 2002; Kim et al., 2008; Pandiyan et al., 2014).

In this study, a second order response surface model was fitted to the data pertaining to sugar consumed and ethanol separately, taking five input variables viz; glucose concentration, xylose concentration, time, yeast extract concentration and peptone concentration. The adequacy of the model was assured through R^2^ and the significance of ANOVA (Table S4).

After optimization of concentrations of inputs like glucose, xylose, yeast extract and peptone, xylose consumption by *S. cerevisiae* LN was estimated to be about 33%. Xylose consumption efficiency ranged from 30 to 50% on mixed substrates by different recombinant/engineered *S. cerevisiae* strains and needed adaptive evolution to enhance xylose utilization. A non-commercial *S. cerevisiae* KE6-12 strain harboring *Schefferomyces stiptis* genes encoding XDH, XR and XK showed only 40–50% xylose utilization in molasses medium supplemented with xylose and had to be pre adapted to improve xylose utilization and ethanol production (Karhumaa et al., 2007).

Rate of glucose consumption was, by and large, not affected during xylose and glucose co-fermentation by recombinant *S. cerevisiae* transformed with sugar transporters from pentose fermenting *Schefferomyces stipitis* and xylose consumption decreased when both sugars were present in medium in equal amounts as compared to fermentations carried out with individual sugars. With 2% initial total sugars (equal amounts of glucose and xylose) in the medium, utilization ranged from 70 to 90% with different transporters (de Sales et al., 2015).

XR and XDH are the key enzymes of pentose metabolism. Ratio of the activities of the same play an important role in the metabolic pathway of the enzymes. Not only the level of XR and XDH activities but the XR: XDH activity ratio is important in xylose metabolism and its diversion toward ethanol formation or xylitol and any other byproduct formation (Träff et al., 2002). In case of recombinant strain of *S. cerevisiae* carrying XYL1 and XYL2 genes from *P. stipitis*, XR:XDH ratios varied from 17.5 to 0.06. Strains with 17.5 ratio formed 0.82 g xylitol g^−1^ xylose consumed, whereas a strain with XR:XDH ratio of 5.0 formed 0.58 g xylitol g^−1^ xylose. Whereas, the strain with low XR:XDH ratio of 0.06 formed no xylitol and less glycerol and acetic acid and produced more ethanol than other strains (Walfridsson et al., 1997; Piotrowski et al., 2014).

To facilitate release of glucose from cellulose contained within lignocellulosic biomasses by enzymes, materials need to be pretreated. However, the high temperatures and chemical conditions generated in these processes lead to the dehydration of glucose and xylose to furfural and hydroxymethylfurfural (HMF), respectively, which are inhibitory to yeast growth and alcohol fermentation. Hydrolysates contain toxic, small molecules arising from residual deconstruction chemicals or biomass-derived inhibitors (Palmqvist and Hahn-Hägerdal, 2000; Field et al., 2015), which impede microbial growth and lead to an increased lag phase of growth and reduced ethanol production at low furan concentrations and cell death at high concentrations. These inhibitors include furans, phenolics and organic acids etc. An industrial strain with innate xylose fermentation ability and inhibitor tolerance would be of significance for industrial bioethanol production.

*S. cerevisiae* LN exhibited good growth in presence of inhibitors like, HMF, furfural and acetic acid (5 g L^−1^). Growth pattern on acetic acid is supported by the fact that weak acids stimulate ethanol production in yeasts. Weak acids can act as uncouplers and stimulate ethanol production in yeasts. Furan and phenolic compounds function as external electron acceptors. The beneficial effect of these compounds is highly concentration dependent and may act synergistically to inhibit yeast growth (Hahn-Hägerdal et al., 2005; Moens et al., 2014) which, in case of xylose fermentation leads to ethanol production. In the current study ethanol was produced by *S. cerevisiae* LN in the presence of furfural (~2.2 g L^−1^) on mixed substrates. Native strains may acquire inhibitor tolerance through adaptation and their tolerance could be enhanced further through short term adaptation during propagation in lignocellulosic hydrolysates improves the inhibitor tolerance of yeast strains also improving their ethanol yield and xylose-fermenting capacity. At low amount, hydrolysates help for developing tolerance, whereas higher amounts decrease cell mass yield during propagation. Certain strains of *S. cerevisiae* isolated from environmental and industrial samples displayed great resistance to furfural, grew and produced ethanol in presence of 3.0 mg mL^−1^ of furfural (Field et al., 2015). They might have adapted to these levels of furfurals while growing in natural habitats where lignocelluloses degradation was ongoing.

## Conclusion

It was observed that the *S. cerevisiae* strain LN had innate capability to utilize xylose and could produce ethanol from mixed substrates. Presence of xylose metabolic pathway in the *S. cerevisiae* strain LN was evident from the XR and XDH sp. activities. The strain was tolerant to inhibitors usually encountered in hydrolysates. It could grow well on biologically and steam pretreated paddy straw hydrolysates to produce ethanol (Arora et al., 2016). Thus, the study showed that xylose assimilation genetic machinery in *S. cerevisiae* LN is not merely artifact of xylose metabolizing pathway which got reduced during prolonged growth on hexoses but it can be optimized by statistical tools and there are prospects of further escalation by adaptive evolution and evolutionary engineering.

## Author contributions

ShS performed all the experiments. EV and KS helped in RSM modeling and statistical analyses of the data. SuS and LN assisted in HPLC analyses. DP provided valuable inputs in writing the manuscript. AA conceptualized the study, analyzed data and wrote the manuscript.

### Conflict of interest statement

The authors declare that the research was conducted in the absence of any commercial or financial relationships that could be construed as a potential conflict of interest.
