# Corrigendum: Augmenting Pentose Utilization and Ethanol Production of Native *Saccharomyces Cerevisiae* LN Using Medium Engineering and Response Surface Methodology

**DOI:** 10.3389/fbioe.2018.00168

**Published:** 2018-11-13

**Authors:** Shalley Sharma, Eldho Varghese, Anju Arora, K. N. Singh, Surender Singh, Lata Nain, Debarati Paul

**Affiliations:** ^1^Division of Microbiology, ICAR-Indian Agricultural Research Institute, New Delhi, India; ^2^ICAR-Indian Agricultural Statistics Research Institute, New Delhi, India; ^3^Amity Institute of Biotechnology, Amity University, Noida, India

**Keywords:** *Saccharomyces cerevisiae*, co-fermentation, inhibitors, hydrolysates, response surface methodology, optimization

In the original article, there was a mistake in Table 2 as published. SE_m_ and CD@5% of ethanol (%) and sugars consumed (g L^−1^) were erroneously exchanged. The corrected Table 2 appears below. The authors apologize for this error and state that this does not change the scientific conclusions of the article in any way. The original article has been updated.

**Table 2:** Growth, sugar consumption and ethanol production by *S. cerevisiae* LN on 2% xylose.

**Time (h)**	**Growth (OD_600 nm_)**	**Ethanol (%)**	**Sugars consumed (g L^−1^)**	**Fermentation efficiency (%)**	**Ethanol yield (g g^−1^)**
24	0.61	0.05 ± 0.18	2.13 ± 0.29	45.11 ± 0.16	0.23 ± 0.003
48	0.85	0.09 ± 0.01	3.74 ± 0.13	46.29 ± 0.46	0.24 ± 0.007
72	1.07	0.11 ± 0.41	3.61 ± 0.19	58.82 ± 0.59	0.30 ± 0.01
^*^SE_m_		0.03	1.11	7.96	0.04
^**^CD@5%		0.10	3.06	21.97	0.11

* SE_m_ denotes Standard error of mean;

** CD@5% denotes critical difference @ 5%.

## Conflict of interest statement

The authors declare that the research was conducted in the absence of any commercial or financial relationships that could be construed as a potential conflict of interest.

