# Supplementary material for: Augmenting Pentose Utilization and Ethanol Production of Native Saccharomyces cerevisiae LN Using Medium Engineering and Response Surface Methodology
Source: Front Bioeng Biotechnol. 2018 Sep 24;6:132. doi: 10.3389/fbioe.2018.00132 (PMC6166573; doi:10.3389/fbioe.2018.00132)
Supplement: Supplementary file 1 [file Table_1.DOCX]

**Supplementary Data**

**Table S1.** Enzyme cocktail for XR activity determination.

| **Solution** | **Volume (µL) added to** | |
| --- | --- | --- |
|  |  |  |
|  | **Control** | **Experimental** |
| **DI water** | 200 | 100 |
| **250 mM Potassium phosphate** | 600 | 600 |
| **100 mM 2-mercaptoethanol** | 100 | 100 |
| **3.4 mM NADPH** | 50 | 50 |
| **0.5 M Xylose** | 0 | 100 |

**Table S2**. Enzyme cocktail for XDH activity determination.

| **Solution** | **Volume (µL) added to** | |
| --- | --- | --- |
|  |  |  |
|  | **Control** | **Experimental** |
| **DI water** | 300 | 200 |
| **500 mM tris-HCl** | 400 | 400 |
| **100 mM 2-mercaptoethanol** | 100 | 100 |
| **4.0 mM NAD^+^**​ | 100 | 100 |
| **1.5 M Xylitol** | 0 | 100 |

**Table S3 (A).** Ethanol production and glucose consumption by *S. cerevisiae* LN through fermentation of 10% glucose with the effect of supplementation

| **Treatment** | **Ethanol produced (g L^-1^)** | | | **Mean** | **Glucose consumed (g L^-1^)** | | | **Mean** |
| --- | --- | --- | --- | --- | --- | --- | --- | --- |
| **Time (h)** | **96** | **120** | **144** |  | **96** | **120** | **144** |  |
| **0.1% (YE+P)*** | 14.19±0.40 | 35.20±0.40 | 19.66±0.17 | 21.18 | 91.79±0.1 | 89.72±0.14 | 89.79±0.60 | 89.02 |
| **0.5% YE** | 19.68±0.16 | 19.19±0.41 | 31.43±0.29 | 23.43 | 100 | 100 | 99.97±0.01 | 99.99 |
| **1% (YE+P)** | 19.06±0.47 | 18.49±0.25 | 20.08±0.46 | 19.213 | 100.00 | 99.98±0.51 | 99.09±0.46 | 99.69 |
| **Mean time** | 17.644 | 22.46 | 23.719 |  | 97.26 | 96.14 | 95.296 |  |
| **SE(Time)** | 5.42 | **CD (Time)** | 11.39 |  | **SE(Time)** | 6.26 | **CD (Time)** | 13.15 |
| **SE(trt)** | 3.13 | **CD (trt)** | 6.57 |  | **SE(trt)** | 3.61 | **CD (trt)** | 7.59 |
| **SE(trt*Time)** | 3.13 | **CD (trt*Time)** | 6.57 |  | **SE(trt*Time)** | 3.61 | **CD (trt*Time)** | 7.59 |

*(YE+P), Yeast extract+Peptone

**Table S3 (B).** Fermentation efficiency and ethanol yield by *S. cerevisiae* LN through fermentation of 10% glucose with the effect of supplementation

| **Treatment** | **Fermentation Efficiency (%)** | | | **Mean** | **Ethanol yield (g g^-1^)** | | | **Mean** |
| --- | --- | --- | --- | --- | --- | --- | --- | --- |
| **Time (h)** | **96** | **120** | **144** |  | **96** | **120** | **144** |  |
| **0.1% (YE+P)*** | 30.96±0.52 | 76.90±0.46 | 43.35±0.32 | 48.307 | 0.16±0.01 | 0.39±0.01 | 0.22±0.01 | 0.24 |
| **0.5% YE** | 38.58±0.71 | 37.63±0.19 | 61.64±0.18 | 45.951 | 0.20±0.01 | 0.19±0.01 | 0.31±0.01 | 0.23 |
| **1% (YE+P)** | 37.38±0.56 | 36.27±0.37 | 39.69±0.65 | 37.78 | 0.19±0.01 | 0.18±0.01 | 0.20±0.01 | 0.19 |
| **Mean** | 35.64 | 47.70 | 48.70 |  | 0.18 | 0.24 | 0.25 |  |
| **SE(Time)** | 12.86 | **CD (Time)** | 27.01 |  | **SE(Time)** | 0.07 | **CD (Time)** | 0.13 |
| **SE(trt)** | 7.42 | **CD (trt)** | 15.60 |  | **SE(trt)** | 0.04 | **CD (trt)** | 0.08 |
| **SE(trt*Time)** | 7.42 | **CD (trt*Time)** | 15.60 |  | **SE(trt*Time)** | 0.04 | **CD (trt*Time)** | 0.08 |

**Table S4.** ANOVA for second order polynomial regression model for response variables

| **Coefficients** | **Product responses** | |
| --- | --- | --- |
|  | **Ethanol** | **Sugar Consumed** |
| **Intercept** | | |
| **β_0_** | 1.152 (0.05) | 79.974 (1.52) |
| **β_1_** | 0.347^ns^ (0.05) | 2.704^ns^ (1.52) |
| **β_2_** | 5.02E-0.5 (0.05) | -2.148^**^ (1.52) |
| **β_3_** | -0.019^**^ (0.05) | 2.334^ns^ (1.52) |
| **β_4_** | 0.040^*^ (0.05) | -1.741^**^ (1.52) |
| **β_5_** | 0.003^*^ (0.05) | -1.088^**^ (1.52) |
| **Interaction** | | |
| **β_AB_** | 0.074^ns^ (0.06) | 1.984^ns^ (1.77) |
| **β_AC_** | 0.084^ns^ (0.06) | 2.570^ns^ (1.77) |
| **β_AD_** | 0.088^ns^ (0.06) | -0.275^**^ (1.77) |
| **β_AE_** | 0.018^*^ (0.06) | -0.953^**^ (1.77) |
| **β_BC_** | 0.070ns (0.06) | -1.349^**^ (1.77) |
| **β_BD_** | -0.130^**^ (0.06) | 3.022^ns^ (1.77) |
| **β_BE_** | -0.054^**^ (0.06) | 0.583^ns^ (1.77) |
| **β_CD_** | 0.043^*^ (0.06) | 3.033^ns^ (1.77) |
| **β_CE_** | 0.005^*^ (0.06) | 1.513^ns^ (1.77) |
| **β_DE_** | -0.079^**^ (0.06) | 0.656^ns^ (1.77) |
| **Quadratic** | | |
| **β_1_** | 0.141^ns^ (0.04) | -4.181^**^ (1.34) |
| **β_2_** | 0.088^ns^ (0.04) | -4.601^**^ (1.34) |
| **β_3_** | 0.015^*^ (0.04) | -1.124^**^ (1.34) |
| **β_4_** | 0.069^ns^ (0.04) | -1.107^**^ (1.34) |
| **β_5_** | 0.048^*^ (0.04) | -1.112^**^ (1.34) |
| **R^2^** | 0.736 | 0.577 |
| **Model Significance** | p<0.001 | p<0.05 |

1, Glucose; 2, Xylose; 3, Time; 4, Yeast Extract; 5, Peptone. * Significant at p < 0.05, ** Significant at p < 0.01; ns: non significant.β_0_, β_1_… βn = Regression coefficients, Figures in parenthesis denotes standard error, R^2^ = R-Square

Figure S1A. Surface plots for ethanol production with respect to time and xylose consumption

Figure S1B. Surface plots for ethanol production wrt glucose and xylose concentration

Figure S1C. Surface plot for ethanol production wrt glucose concentration and time

Figure S1D. Surface plot for ethanol production wrt glucose and yeast extract concentration

Figure S1E. Surface plot for ethanol production wrt glucose concentration and peptone concentration

Figure S1F. Surface plot for ethanol production wrt peptone and time

**Figure** S**1.** Surface plots for ethanol production with respect to xylose, glucose, yeast extract, peptone concentrations and time (A-F) respectively

Figure S2A. Surface plot for sugar consumption wrt the concentrations of xylose and glucose

Figure S2B. Surface plot for sugar consumption wrt xylose concentration and time

**Figure** S**2.** Surface plot for sugar consumption with respect to glucose, xylose concentrations and time
